# Epidemiological profile of the suicidal in the third poorest state of Brazil

**DOI:** 10.1192/j.eurpsy.2021.2194

**Published:** 2021-08-13

**Authors:** G. Lessa De Souza Maia, M. Melo Gomes Do Nascimento

**Affiliations:** Medicine, Centro Universitário Tiradentes, Maceió, Brazil

**Keywords:** Suicide, epidemiology, Brazil, causa mortis

## Abstract

**Introduction:**

Alagoas is one of the poorest states of Brazil and its HDI is the country’s worst.

**Objectives:**

Present the epidemiological profile of suicides that occurred in the State of Alagoas from 2008 to 2018.

**Methods:**

This research is epidemiological, descriptive and transversal. In this sense, data from the Universal Health System Informatics Department (DATASUS) were used to analyze the age range, marital status, race, sex, education and cause of death of the suicide victims.

**Results:**

1245 people committed suicide in Alagoas in the period of 10 years, they were 951 men (76%) and 294 women (24%). The main cause of death was self-harm caused by hanging, strangulation and suffocation (ICD X-70), occurring in approximately 67% of cases (836 people), followed by self-poisoning by drugs and medication (ICD X-64) 140 people, and pesticides (CID X-68) 92 people. The auto injuries caused by firearms (ICD X-72 to X-74) totalled 45 victims, while the self-inflicted injuries intentionally caused by precipitation from a high place (ICD X-80) totalled 38 victims. Most of those who took their lives were single (57%), brown (88%), had between 15 and 39 years old (55%) and did not have their education level informed (75%).

**Conclusions:**

Thus, the present study demonstrated that there is a compromise of important statistical data on education level and there is the inexistence of data on family income and sexual orientation, which may help to understand the phenomenon of suicide in Alagoas. Despite all this, it was possible to identify a group of risk for suicide in the State: brown, single and young men.

**Disclosure:**

No significant relationships.

